# Scraps

**Published:** 1893-12

**Authors:** 


### SCRAPS

PICKED UP BY THE ASSISTANT-EDITOR.

---

**Christmas Bills.**—The following appears at the foot of a bill-head of a Kansas physician. It is unique, original, and pointed, and we presume effective: "A prompt settlement of this bill is requested. If bills are paid monthly, a discount of ten per cent. is given. Bills not paid promptly will be passed to my attorney for collection. If you pay your physician promptly he will attend you promptly, night or day, rain or shine, while your slow neighbour suffers and waits, as he made the doctor wait, and while he is waiting the angels gather him in."—*Kansas Medical Journal.*

**Clinical Records** (5).—A writer in a journal narrates the following experience: I was called out one morning at 3 to see a lady who was "very ill." Upon arriving at the house I was shown into a room where a lady was sitting in a composed attitude. I at once asked her where I was to find my patient. She replied with calm deliberation, "I am your patient." "Indeed. What is the matter?" "If you will take a chair," said the lady, "I will tell you. The fact is that I fear I have taken to drinking." "I am very sorry to hear it. When did you make the mistake?" "I am not quite confident as to dates, but as well as I can remember it was in the year 1866—early in 1866." "But do you consider it was necessary to knock me up at 3 a.m. in 1893 to see you because you took to bad habits in 1866?" "I thought that was the wisest course to pursue."

**Medical Philology** (VIII.)—In the *Catholicon Anglicum* is this definition:

"a **Blossom**; *colloq*ui*ntida, q*ui*nticie.*"

Mr. Herrtage's note is:

Ducange gives "*Colloquintida.* Colocynthis; *coloquinthe,*" and Cotgrave renders "*Coloquinthe*" by "the wilde and flegme-purging Citrull *Coloquintida.*" Cooper has "*Colocynthis.* A kynde of wylde gourdes purgeyng fleume, called *Coloquintida.*" "*Colloquintida: genus herbe amarissime,* i.e. *cucurbita. Quintecie,* Blosmes." Medulla.

The *Catholicon* extract is not given in the *New English Dictionary.* No explanation has yet been given why in the *Catholicon* and the *Medulla* the general term "Blossom" should be applied to one particular plant. "Blossom," in this sense, is one of the words of which Mr. Herrtage was unable to obtain any satisfactory explanation. In searching in the few books to which I have had easy access, I have only succeeded in getting into greater difficulty.

In a glossary, apparently of the eleventh century (MS. Cott., Cleopatra A, III., printed in Wright's *Vocabularies*, 2nd Ed.), "Ligustra" is given (437, 17) as the Latin equivalent of "blostman," which was one of the Early English forms of the plural of blossom. Ligustrum, which is commonly rendered by modern translators as privet, is used by Virgil (E. II. 18), Ovid (M. XIII. 789), and Martial (I. cxvi., 3) to emphasise the idea of excessive whiteness. But "ligustrum," like lotus, seems to have been a term applied to various plants. In vocabularies dated from the 8th to the 11th century, which are given by Wright, it will be found as "hunigsuge" (30.12, 517.35) or "hunisuge" (139.6, 433.2). In the middle of the 13th century it appears as "triffoil" or "hunisuccles" (558.15). In the 15th century it signifies a "primerose" (592.41 and 712.18), and "a cowslope" (713.11) or "a cowyslepe" (786.24). Mr. Cockayne in *Saxon Leechdoms* (III. 332) gives "Hone sokel, *Honey suckle;* any plant from which honey may be sucked. 1. *Melilotus*, MS. Bodl. 536. 2. *Trifolium pratense*, Laud. 553, and still in use. 3. *Lonicera periclymenum.*" To make confusion worse confounded, we find in an eleventh century vocabulary (Wright, 364.31) "*Colocinthidæ*, hundescwelcan," which latter word is explained by Mr. Cockayne (*op. cit.*, III. 333) as berries of the wayfaring tree, "baccæ de viburno opulo," and he cites from the Gl. Harl. 3388, *Jarus*

*amarus*, which there has the English meaning of "hundes quelke." A fifteenth centary Vocabulary (Wright, 588.29) describes *Jarus* as Cokkupyntel or Calvysfote, the old names of the *Arum maculatum*. If any reader will kindly unravel this tangle, I shall be grateful.

That colocynth had for a long period a very great reputation for many virtues in addition to its aperient powers may be seen from the following quotations:

> It cleanses the brain, the nerves, the muscles, the chest and the lung . . . it prevents the flowing of water down from the eyes, and is a wonderful thing for asthma and chronic cough.—Translation of a passage in Matthioli's Commentary upon Dioscorides, 1563, p. 632.
>
> But Coloquintida is not only good for purgation, it is a remedie for . . . the falling sicknesse, the stuffing of the lungs.—Gerarde, *Herbal*, 1597 pp. 769–70.
>
> 'Tis hot and dry in the third degree; it purgeth phlegme and other grosse and clammy humours from the deepest and most remote parts of the body, as brain, nerves, muscles, joynts, lungs, breast, and womb especially, for which cause it is used for the inveterate pains of the head and hemicranies, apoplexy, falling-sicknesse, megrim, or swimming of the head, asthma, coughs, difficulty of breathing, cold diseases of the joints, and wind colick, to scour glassy phlegme from the intrals; it hurts the stomach and guts much by sticking to the filmes of them, and is hurtfull for children, old folks, and women with child.—*The Expert Doctor's Dispensatory*, 1657, pp. 349–50.

Shakspere knew, perhaps from persoual experience, the taste of the drug. Iago says (*Othello* I. iii. 354–5), "the food that to him now is as luscious as locusts shall be to him shortly as bitter as coloquintida."

**Preventive Medicine.**—Our sixteenth and seventeenth century forefathers were alive to the desirability of taking steps to prevent disease. Two instances which I have recently noticed are worth quoting. They illustrate different phases of the importance of prophylaxis.

Here are the counsels which in William Bullein's *Dialogue against the Fever Pestilence*, (1578) Medicus gives his rich patient, Antonius, for avoiding the plague; many of them would still be considered excellent, but some have been improved upon.

> "First of all, let all men, women, and children auoide oute of the euill ayre into a good soyle, and then, accordyng to their age, strength of nature, and complexion, let euery one of them with some good medicine drawe from the bodie superfluous moysture, and diminsh humour, hotte and drie, and vse the regiment of diet to driyng sharped with vineger or tart thynges, and lesser meates; not so much wine as they haue vsed in custome; neither Potage, Milke, vnripe fruites, hotte Spices, Dates, or Honie, or sweete meates, wine with Suger, are not tollerable; no anger or perturbations of the mynd, specially the passion called feare, for that doth drawe the spirites and blood inwards to the hart, and is a very meane to receiue this plague; neither vse actes venerous, nor bathyng, either with Fume, stoue or warme water, (for this cause)—they all doe open the pores of the body; neither quaffyng or muche drinkyng. Euen so thirste or drinesse is not tollerable, or immoderate exercise or labour, specially after meate. Music is good in this case, and pleasaunt tales, and to haue the meates well sauced with cleane sharpe vineger. Forget not to keepe the chamber and clothyng cleane, no priuies at hand, a softe fire with perfumes in the mornying. Shifte the lodging often time, and close in the Southeaste windowes, specially in the tyme of mistes, cloudes, and windes; And vse to smell vpon some pleasaunt perfume; And to bee letten bloud a little at once, and to take Pilles, *contra pestem*, that is a good preseruative against the plague."

In 1639 was published *The Charitable Physitian*, which I. W. translated into English from the French of Philbert Guibert, Esquire, Doctor Regent in the faculty of Physick in Paris. It was compiled for the purpose of "shewing the manner to make and prepare in the house with ease and little paines all those remedies which are proper to all sorts of diseases, according to the advice of the best and ordinariest Physitians." It concluded its list of such remedies with this "Charitable and notable advertisment to the publike":

> It is necessary for all sorts of people to keepe by them a syringe or bladder, and pipe to give Clysters, and to make or cause to bee made the said Clysters in their houses; for what disease soever hapneth or ariveth there, is nothing so proper at the first as a Clyster; but if your servant or any other unto you belonging should give a Clyster to one sicke of the Plague, Poxe, Measells, Purples, Dissentery, small Poxe, Vlcers, Sores, Boyles, or any other pestiferous disease; or should lend it to any that should doe the like, and come and give you a Clyster with the same pipe without washing and cleansing, any of the said diseases would be upon you in lesse then an houre after, and to all your family; therefore see the pipe well warmed, washed and cleansed, before you take the said Clyster, and lend your pipe to none but to those that you know very well.

